# Brushing RemInder 4 Good oral HealTh (BRIGHT) trial: does an SMS behaviour change programme with a classroom-based session improve the oral health of young people living in deprived areas? A study protocol of a randomised controlled trial

**DOI:** 10.1186/s13063-019-3538-6

**Published:** 2019-07-23

**Authors:** Zoe Marshman, Hannah Ainsworth, Ivor Gordon Chestnutt, Peter Day, Donna Dey, Sarab El Yousfi, Caroline Fairhurst, Fiona Gilchrist, Catherine Hewitt, Claire Jones, Ian Kellar, Sue Pavitt, Mark Robertson, Sarwat Shah, Katherine Stevens, David Torgerson, Nicola Innes

**Affiliations:** 1 School of Clinical Dentistry, University of Sheffield, Claremont Crescent, Sheffield, S10 2TA UK; 2 York Trials Unit, Department of Health Sciences, Faculty of Sciences ARRC Building, University of York, York, YO10 5DD UK; 3 Cardiff University School of Dentistry, Heath Park, Cardiff, CF14 4XY UK; 4 School of Dentistry, University of Leeds, Leeds, LS2 9LU UK; 5 School of Education and Social Work, University of Dundee, Nethergate, Dundee, DD1 4HN UK; 6 Health information Centre, University of Dundee, (Main Level 5 Corridor), Second Floor (Level 7), Mail Box 15, Ninewells Hospital & Medical School, Dundee, DD1 9SY UK; 7 School of Psychology, University of Leeds, Lifton Place, Leeds, LS2 9JT UK; 8 Dental Translational & Clinical Research Unit, School of Dentistry, University of Leeds, Leeds, LS2 9LU UK; 9 School of Dentistry, University of Dundee, Park Place, Dundee, DD6 8EF UK; 10 Department of Health Sciences, Faculty of Sciences ARRC Building, University of York, York, YO10 5DD UK; 11 Health Economics and Decision Science, School of Health and Related Research, The University of Sheffield, Regent Court, 30 Regent Street, Sheffield, S1 4DA UK

**Keywords:** Dental caries, Caries prevention, Prevention, Behaviour change, Randomised controlled trial, Child dental health, mHealth, Short messaging service

## Abstract

**Background:**

Almost one-half of 12–15 year olds living in deprived areas of the UK have dental caries (tooth decay) with few oral health promotion programmes aimed at children of this age. Mobile phone-based interventions such as short messaging service (SMS) interventions have been found effective at changing certain behaviours and improving health outcomes. This protocol describes the BRIGHT Trial, investigating the clinical and cost-effectiveness of a behaviour change intervention—classroom-based session (CBS) embedded in the curriculum and a series of SMS delivered to participants twice daily to remind them to brush their teeth, compared to usual curriculum and no SMS—to reduce the prevalence of dental caries in young people from deprived areas.

**Objectives:**

To investigate the clinical and cost-effectiveness of a complex intervention to improve the oral health of young people living in deprived areas.

**Methods/design:**

This is a school-based, assessor-blinded, two-arm cluster-randomised controlled trial with an internal pilot trial. Overall, the trial will involve approximately 5040 11–13 year olds in 42 schools with a 3-year follow-up. The trial will take place in secondary schools in England, Scotland and Wales. The primary outcome is the presence of carious lesions in permanent teeth at 3 years. Secondary outcomes are: number of carious teeth, frequency of twice-daily toothbrushing, plaque levels, gingivitis, child health-related quality of life and oral health-related quality of life. A cost-utility analysis will be conducted.

**Discussion:**

The findings of the trial have implications for embedding oral health interventions into school curricula guidance produced by national bodies, including departments for education and dental public health and guideline-development organisations.

**Trial registration:**

ISRCTN registry, ISRCTN12139369. Registered on 10 May 2017.

**Electronic supplementary material:**

The online version of this article (10.1186/s13063-019-3538-6) contains supplementary material, which is available to authorized users.

## Background

Untreated dental caries (tooth decay) is the most prevalent condition worldwide, affecting 2.4 billion people [1]. The consequences for children include pain [2], loss of sleep, problems with eating and speaking and time off school [3–5]. Dental caries has a significant impact on young people’s daily lives with around 50% of 12–15 year olds reporting toothache and around one-quarter of 12–15 year olds reporting difficulty eating [4]. Dental caries can also affect the general health and quality of life of children, interfering with nutrition, school attendance and school performance [6–8]. A recent systematic review found children with one or more decayed teeth had a higher probability of poor school performance and poor school attendance than children free of obvious caries [8].

Dental caries affects an average of one in three 12 year olds in the UK and although it affects all parts of society, it shows a positive, linear association with deprivation [9–11]. In 2013 in England, 32% of 12 year olds experienced dental caries and required treatment, ranging from 46% of those eligible for free school meals (FSM) to 30% of those ineligible. For 15 year olds 44% required treatment, 59% of those eligible for FSM and 43% of those ineligible [4]. Treating oral diseases is expensive, costing NHS England £3.4 billion annually. Children’s tooth extractions alone, carried out under general anaesthesia, and as a result of dental caries, costs an estimated £36 million annually in England [12].

The use of fluoridated toothpaste is considered to have been largely responsible for the dramatic reduction in the levels of dental caries from a mean of 8.4 decayed, missing and filled teeth (DMFT) in 1973 [13] to 1.4 in 2013 [4]. Brushing with fluoridated toothpaste is one of the most highly effective preventive measures [14, 15]. Observational studies have shown current levels of efficacy, frequency and duration of toothbrushing to be inadequate [16–18], increasing the risk of dental caries [9].

Mobile health (mHealth) describes multimedia technologies that interface with healthcare delivery and are supported by mobile devices, almost exclusively mobile phones [19]. In 2017, 86% of 12–15 year olds in the UK owned a mobile phone [20], providing the potential to deliver large-scale health behaviour change interventions. While young people of lower socio-economic status are subject to inequality in access and use of health services, research suggests they have equivalent mobile phone access to their more affluent peers [21]. Short messaging service (SMS) are short text messages sent from computers, phones or other mobile devices usually to phones and are the most widely studied mHealth interventions [19, 22].

A recent systematic review of preventive health behaviour change SMS interventions found a small but statistically significant weighted mean effect size for the impact of SMS on preventive health behaviour change (d = 0.24) with positive effect of SMS interventions in 11 of the 35 included studies, with a further 13 studies having mixed effects [23]. The key features of SMS interventions include duration, tailoring, targeting of the content and how SMS are used along with other activities [23]. The duration of interventions typically ranged from 1 to 66 weeks with a median duration of 12 weeks. There was some suggestion that interventions lasting 6–12 months were associated with greater effects than shorter interventions. The frequency of messages varied from five times per day to once a month depending on the expected frequency of the targeted behaviour [23]. The limitations of existing studies included a lack of a specified theoretical framework, insufficient power to detect change, only short-term follow-up after the end of the intervention, low retention rates and failure to blind assessors [23–25]. It was recommended that future studies ensured the intervention, including the SMS, was developed rigorously, the SMS were written to be appropriate for the target population and the SMS were tailored to individuals according to their age and gender and used the participant’s name.

Although the mobile phone has been investigated as a vehicle for health behaviour change using SMS interventions, there is a paucity of research with adolescents and involving oral health behaviour change [26]. One recent study, of unemployed young people aged 18–24 years in New Zealand, investigated the Keep on Brushing (KOB) programme of weekly SMS and free toothbrushes/toothpaste, seeking to boost motivation [27, 28]. The KOB intervention was underpinned by the Health Belief Model [29]. This study was conducted in a branch of the New Zealand Government’s employment and beneficiary services and 171 participants were recruited and completed a baseline survey and then received a series of motivational SMS over 10 weeks. Self-reported toothbrushing frequency was the primary outcome measure. Other socio-demographic data (age, gender, ethnicity, employment status) and method-specific (level of attrition, distribution of successful text messages deliveries, active withdrawal) variables were also collected. Self-reported toothbrushing of twice or more per day increased from 51% at baseline to 70% at week 3, 74% at week 6, and 73% at week 9. No important differences were noted between ages, gender or ethnic groups, although attrition was relatively high with only 26% participating by week 9. The authors concluded that motivational SMS improved the self-reported oral health of this hard-to-reach group and suggested a randomised controlled trial including a longer intervention with tailoring of the messages was needed.

The aim of this study is to establish the clinical and cost-effectiveness of a SMS behaviour change programme to improve the oral health of young people living in deprived areas.

The main trial will:

1. investigate the effect of the intervention on caries prevalence
2. investigate the effect of the intervention on twice-daily tooth brushing, oral health-related quality of life and oral health behaviours
3. investigate the cost-effectiveness of the intervention
4. explore implementation, mechanisms of impact and context through a process evaluation

## Methods/design

### Study design

The BRIGHT trial is a multi-centre, school-based, assessor-blinded, two-arm cluster-randomised controlled trial (RCT).

The population being investigated are pupils in schools with above average percentage of pupils eligible for free school meals (FSM). The BRIGHT intervention is based on the New Zealand KOB study intervention. It is a multi-component, complex intervention with two parts: 1) a short classroom-based session (CBS) embedded in the curriculum; and 2) a series of follow-up twice-daily SMS. Pupils in the control group will continue to receive routine education and no text messaging, and the primary outcome is the presence of caries.

### Procedure

#### Study setting

The trial aims to recruit 5040 young people, aged 11–13 years (year 7 and year 8 in England/Wales; S1 and S2 in Scotland) from 42 schools across Scotland, England and Wales with above the national average percentage of pupils eligible for FSM. These year groups have been chosen purposefully to minimise disruption to English and Welsh GCSE and Scottish Qualifications Authority National 5 exam years, and also to confine 3-year follow-up to within the school setting to avoid the need to follow participants to further education settings.

#### School eligibility (inclusion/exclusion criteria)

To be eligible for participation, schools must:

- be located in Scotland, England (South Yorkshire and West Yorkshire), or South Wales
- be state funded
- have pupils aged 11–16 years old
- have at least 60 pupils per year group
- have above the national average percentage (for each devolved nation) of pupils eligible for FSM

Schools will be ineligible for inclusion if they are:

- in Special Measures where the school is judged by the Office for Standards in Education, Children’s Services and Skills to be failing, or likely to fail, to provide an acceptable standard of education
- due to close

Eligible schools will be identified based on data from the Department for Education’s register of educational establishments in England and Wales and Education Scotland. Schools will be approached by local research teams and invited to take part.

#### Participants

Participants are young people aged 11–13 years old at baseline and attending schools with above the national average percentage of pupils eligible for FSM.

#### Young person (participant) eligibility (inclusion/exclusion criteria)

Young people will be eligible for inclusion if they are:

- a pupil at a participating school, and
- aged 11–12 years (in year 7 in England/Wales or S1 in Scotland) or 12–13 years (in year 8 in England/Wales or S2 in Scotland)

Young people will be ineligible for inclusion if:

- they have no functioning mobile telephone of their own, or
- their parent/carer does not want them to be part of the trial and they opt out

#### Young person (participant) recruitment

Recruitment strategies have been based on consultation with young people through a youth organisation particularly concerned with hard-to-reach young people (Children and Young People’s Empowerment Project (Chilypep)), teachers and head teachers, a school welfare officer and school nurse and from learning during the internal pilot trial. A young person forum has contributed to the design of the trial and successfully ran throughout the internal pilot trial. This forum will continue to advise on participant recruitment and the best ways of optimising continued engagement with hard-to-reach pupils during the trial.

#### Consent procedure

Parents/carers will have the opportunity to state that they do not want their child to participate (opt out), by completing and returning an opt-out form to their child’s school. Eligible young people whose parents/carers have not opted them out of the research will then be invited to take part in the trial. The young people who agree will be asked to sign a consent form by members of the local research team or teachers.

All young people who complete the baseline questionnaire and dental assessment will be given a £10 voucher to thank them for their time; all young people who complete the follow-up questionnaire and dental assessment will be given a £5 voucher as a thank you.

#### Study procedure

##### Randomisation

Allocation will take place within schools by randomising schools 1:1 to one of two regimes:

1. 11–12-year-old pupils (year 7 in England and Wales/S1 in Scotland) to receive the intervention and 12–13-year-old pupils (year 8 in England and Wales/S2 in Scotland) to act as the control group, or
2. 12–13-year-old pupils (year 8 in England and Wales/S2 in Scotland) to receive the intervention and 11–12-year-old pupils (year 7 in England and Wales/S1 in Scotland) to act as the control group

An allocation sequence, stratified by school using blocks of size two [30], will be generated by an independent statistician. This sequence will be retained by the statistician and will not be accessible to other members of the trial research team. Once a school is ready to be randomised, following collection of their baseline data, the year groups within that school will be randomised by assigning their year 7 and year 8 cohorts (in that order) to the next block in the allocation schedule. The research team and schools will then be informed of which year group has been allocated to receive the BRIGHT intervention and which has been assigned to the control arm. Since the statistician performing the allocation and the teams recruiting the schools are completely separate, and the allocation schedule cannot be known to anyone but the statistician in advance of randomisation, allocation concealment is assured.

##### Blinding

Given the nature of the intervention, it will not be possible to blind schools or participants (pupils) to their group allocation; however, clinical examinations will be performed by a trained and calibrated dentist blind to the allocation of the pupils, as far as possible. It is possible that pupils may unblind the assessors but they will be encouraged not to discuss the intervention with the dental team to minimise this risk. We shall ask the dental teams to record whether they became unblinded to the pupil’s allocation.

### Intervention and comparison (control)

The aim of the intervention is to increase toothbrushing frequency with a fluoride toothpaste and thereby reduce the likelihood of the development of dental caries. The intervention consists of two components: (1) a CBS delivered by teachers in the schools’ curricula followed by (2) a series of twice-daily SMS to mobile phones. The intervention meets the Medical Research Council definition of a complex intervention in terms of the interactions between components and the difficulty of behaviours required by those receiving the intervention [31]. The control group will not receive the CBS or SMS messages. The KOB intervention has been refined to be more acceptable to young people and informed by recent behaviour change theory [32].

#### CBS

Teachers will deliver the single CBS (50 min in duration) in the school environment to participants in the intervention arm. The schools will receive a teacher’s guide that will outline the learning intentions and success criteria for the lesson, in addition to the appropriate teaching resources in order to deliver the lesson. The lesson has been quality assured in England, Scotland and Wales. The number of schools that report delivering the CBS will be reported.

#### SMS

The schedule of SMS was developed using young people’s own words and will reinforce the messages from the CBS. SMS will be provided via mobile phones twice daily according to the recommended frequency of toothbrushing with a fluoride toothpaste. Participants will be reminded to inform the study team of any changes to their mobile phone number. When participants wish to stop receiving text messages, they can text STOP for free at any time. Messages will also be re-started on request. The number of SMS messages received by the pupils will be summarised descriptively.

### Outcome measures

#### Primary outcome

The primary outcome for this trial is the presence of at least one treated or untreated carious lesion in any permanent tooth measured at the young person-level using the permanent tooth index 'DMFT' (Decayed, Missing and Filled Teeth) where decay is measured as carious lesions extending into dentine - International Caries Detection and Assessment System (ICDAS) levels 4-6 [33], at three years follow-up. The outcome will be measured cross-sectionally at 3 years, and is regardless of the presence or absence of caries in the teeth at baseline.

#### Secondary outcomes

- Caries (D_4–6_ MFT) at 2 years: the presence of at least one treated or untreated carious lesion into dentine in permanent teeth (ICDAS levels 4–6) at 2 years follow-up.
- Caries (D_1–6_ MFT) at 2 and 3 years: the presence of at least one carious lesion in permanent teeth (ICDAS levels 1–6) at 2 and 3 years follow-up.
- Number of carious teeth at 2 and 3 years: The number of permanent teeth with any treated or untreated carious lesions (ICDAS 1-6, and caries into dentine 4-6) at 2 and 3 years.
- Twice-daily toothbrushing: self-reported toothbrushing frequency using validated questions from the national Child Dental Health surveys at baseline, 6 months and 1, 2 and 3 years. To validate the self-reported measure, two proxy clinical objective indicators will be collected: (i) clinically assessed plaque levels using Turesky’s modification of the Quigley Hein Plaque Index [34, 35]; and (ii) clinically assessed gingivitis using gingival bleeding (modification of the Gingival Index of Löe) [36] and mean number of bleeding gingival sites per child. The clinical measures will be carried out at baseline and at the end of years 2 and 3.

#### Other outcomes

- Health related quality of life (HRQoL) will be assessed using the Child Health Utility 9D (CHU9D) [37]. This will be measured at baseline and at years 1, 2 and 3.
- Child oral HRQoL will be assessed using the CARIES-QC. This will be measured at baseline and at years 1, 2 and 3.

Oral health behaviours will be assessed based on self-reported data from young people using questions from the national Child Dental Health Survey [4, 38] on diet, use of dental services and other forms of fluoride use, which will allow assessment of confounding. This will be measured at baseline, 6 months and at years 1, 2 and 3.

For cost-effectiveness, health service resource use will be assessed for the health economic analysis based on data reported by parents via a questionnaire. This will be measured at baseline and at years 1, 2 and 3. Resource use may also be estimated from routine data sources.

Impact on school attendance will be measured by asking schools to provide the attendance record of all participating young people at baseline and at 1, 2 and 3 years.

The impact of the intervention on young people from deprived areas specifically will be assessed. Young people’s eligibility for FSM will be collected from their school and Income Deprivation Affecting Children Index (IDACI) scores will be calculated where possible from participant’s home postcodes.

A mixed method process evaluation will also be conducted to explore implementation, mechanisms of impact and context of the complex intervention [39].

**Fig. 1:**
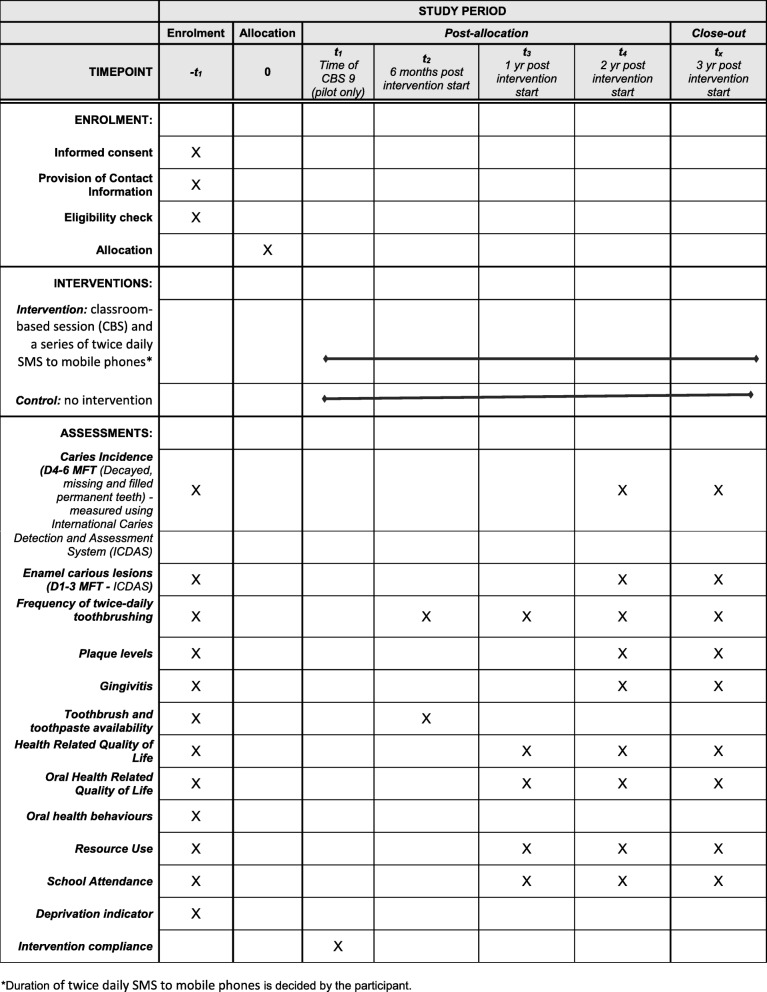
Schedule of enrolment, interventions and assessments

Figure 1 describes the schedule of recruitment, assessments and intervention delivery.

#### Data collection

Data collection will be carried out in the secondary schools under standard dental epidemiological conditions and with questionnaires completed by young people in school time and by parents at home.

The University of Dundee and York Trials Unit (YTU) at University of York will act as the data controllers for this study. All information collected during the course of the trial will be kept strictly confidential. YTU and the regional sites will comply with all aspects of the General Data Protection Regulation 2016 applicable in the UK from May 2018. Personal data will be processed under Article 6 (1) (e) (*Processing necessary for the performance of a task carried out in the public interest*) and Special Category data under Article 9 (2) (j) (*Processing necessary for ... scientific ... research purposes)* of the General Data Protection Regulation 2016. Data sharing agreements will be put in place with participating schools.

A unique trial identification number (trial ID) will be generated for each participant, details of which will be entered into the trial management system. All data, from baseline through to final follow-up, will be collected on paper using case report forms (CRFs) and identified solely by the trial ID; they will be scanned at YTU using Teleform data capture software into a bespoke data management system. Both the trial management system and the data management systems are held on secure University of York servers with access limited to specified members of YTU staff. The paper consent forms and paper CRFs will be held separately and securely in a controlled access area in locked cabinets.

A young person’s mobile phone number, along with their nickname (to which text messages will be addressed) and text message time preference, will be uploaded by YTU directly to the Health Informatics Centre (HIC), University of Dundee. No other details will be uploaded. HIC Services operates a secure Safe Haven environment with strong data governance for the provisioning of data.

##### Sample size

The estimated proportion of UK 12 year olds with caries is 32%, with estimates of 46% for those eligible for FSM and 30% for those not eligible for FSM [4, 40]. Based on a systematic review of interventions for caries prevention to increase the frequency of toothbrushing [41], an absolute reduction in the proportion of young people with caries of 8% might be expected with this intervention. An individually randomised trial powered at 90% (5% two-sided α) to detect a reduction in the proportion of young people with caries from 32% to 24% would require a sample size of 1320. Since this is a cluster trial, this figure needs to be inflated to account for the correlation of the outcomes within schools, as measured by the intraclass correlation coefficient (ICC). Few estimates of a school-level ICC are available for dental data. In a previous study evaluating a behaviour change programme for preventing dental caries in primary schools, an ICC of 0.01 was used, which was estimated using their own unpublished data [42]; a more conservative ICC of 0.02 has been chosen for this trial.

Additionally, since randomisation is taking place at the level of the year group, and not at the school-level, there is the potential for some contamination (e.g. pupils in the year group assigned to the control arm receiving information about the intervention from pupils in the other participating year group assigned to receive the intervention). There is an argument to therefore increase the sample size further to account for some level of contamination, which has the potential to dilute the treatment effect. We have assumed only partial contamination effects (i.e. those contaminated gain half the treatment benefits as it is unlikely that any pupil in the control group would receive all the intervention) for 27% of the control group pupils (based on findings from the internal pilot phase of the trial). In total, 40 schools are required assuming within-school (year group level randomisation), an average of 60 pupils per year group, an ICC of 0.02, and 20% pupil-level attrition at follow-up. This would give us 90% power (5% two-sided α) to detect an 8% absolute reduction, from 32% to 24%, in the proportion of pupils with caries. An attrition rate of 20% was estimated as the pupils will continue to be at school for the duration of the trial so it will be possible to reach and engage with them through the school and in an environment they recognise as educational and supporting their interests. Overall, 42 schools will be recruited to allow for the potential that a small number of schools may withdraw.

### Data analysis

All analyses will be conducted on an intention to treat basis, including all randomised young people in the groups to which their year group was allocated irrespective of deviations based on non-compliance, unless otherwise stated. Data from the internal pilot will be used in all analyses.

The primary analysis will compare the proportion of young people with any treated or untreated caries in permanent teeth at 2 and 3 years between the intervention and control groups using a repeated measures binary logistic multilevel model, with the primary time-point of interest at 3 years. The model will control for presence or absence of caries at baseline, year group, time and an interaction between treatment and time as fixed effect covariates. Pupils and school will be included as random effects (to allow for clustering of data within each pupil (over time) and school). Cohen’s kappa coefficient will be used to measure the intra-examiner agreement of presence of carious lesions at ICDAS code 4–6 for 5% of participants who will be examined twice at a particular time point.

A subgroup analysis will be conducted looking at participants with baseline caries by including presence or absence of caries at baseline in an interaction with treatment group in the primary model. The hypothesis is that young people with caries at baseline are more likely to have caries at follow-up than those who do not have caries at baseline.

Secondary analyses will compare self-reported twice-daily brushing frequency at 6 months and 1, 2 and 3 years using a repeated measures binary logistic multilevel model. Continuous measures (Plaque Index Gingival Index of Löe), mean number of bleeding gingival sites per child and CARIES-QC will be analysed using a covariance pattern model. Other secondary outcomes will be analysed using appropriate regression techniques.

### Health economic assessment

A cost-utility analysis will be conducted. This will estimate the mean differences in costs and quality adjusted life years (QALYs) and report the incremental cost-effectiveness ratio (ICER) for each pathway. The cost-utility analysis will be conducted in line with current recommendations from NICE. In particular, an NHS and Personal Social Services perspective will be taken for costs, and health benefits will be quantified using QALYs. The longer term cost-effectiveness will be modelled to estimate the longer term resource use and HRQoL implications of the intervention.

QALYs will be estimated using the CHU9D [37] reported at baseline and annually thereafter. The CHU9D will be valued using published population tariff values [37, 43], allowing QALYs to be estimated for each arm using the trapezium rule to calculate the area under the curve.NHS resource use will be measured for each participant at baseline and annually up to 36 months. This will include all medication costs (e.g. antibiotics) and visits to dental practices for treatment and health services (e.g. referral to specialists in paediatric dentistry, dental admission for a general anaesthetic) using the parent resource use questionnaire.

### Serious adverse events and adverse events

All participants in the BRIGHT trial will have a dental assessment and complete questionnaires throughout the study period. The intervention participants will receive a CBS about oral health and text message reminders about toothbrushing. Due to the nature of participant involvement, no serious adverse events or adverse events are anticipated that will be unexpected and related.

However, the following procedures will be in place to seek to capture any complications associated with the trial:

- Young people and parents/carers will be informed in the participant information sheet that they are able to report any concerns or anything out of the ordinary that has happened to them as a result of taking part in BRIGHT to the research team during the course of the study. Contact details are provided.
- The dental examination case report form will provide space for the dental examiner to record any suspected serious pathologies, safeguarding issues or unexpected and related adverse events or serious adverse events identified at the time of the dental assessment.

The BRIGHT trial team will monitor incoming data in response to these questions.

### Expected events

It is expected that some participants may experience non-serious adverse events such as minor discomfort in their jaw as a result of keeping their mouth open during the dental assessment, similar to that experienced during a check-up at the dentist. It is also possible that some minor bleeding from the gums might occur as a result of checking for the presence of dental plaque during the clinical examination.

It is also expected that there may be unrelated incidents of hospitalisations, illnesses, disabling/incapacitating/life-threatening conditions, other common illnesses and rarely deaths in the study population; we will not seek to record all such events. We only seek to record those that could be related and unexpected.

### Reporting adverse events

Details of any serious adverse events or adverse events reported by the participants will be considered by the principal investigators and research team. Only details of any serious adverse events that are required to be reported to the Research Ethics Committee, i.e. events which are related to taking part in the study and are unexpected, will be recorded using a trial adverse event form. The adverse event reporting period for this trial begins as soon as the participant consents to be in the study and ends at the final data collection point.

### Suspected serious pathology

In the very rare circumstance that a serious dental/oral issue (e.g. oral cancer, gross swelling or sepsis) is identified during the clinical assessment, dental assessors will contact the Chief Investigator or Co-Principal Investigator, who will (in line with good practice) discuss with a second colleague to decide on the most appropriate person for the child to be referred to. If it is agreed that the young person should be referred to someone else, then the school will be contacted, and we will work with the school and the school nurse to ensure that the young person reaches the appropriate help, whether that is a health or social care professional.

### Publication and dissemination policy

The study will inform on the cost-effectiveness of a low cost SMS delivered alongside a classroom-based intervention for secondary schools by local authorities or departments of education to reduce dental caries in young adults. The results will be published in a NIHR HTA monograph and high impact, peer reviewed dental journals and in education academic journals and newsletters. The results will be presented at international and national dental and education conferences. The findings will also be disseminated to the wider public health and education audiences. A publication policy has been developed.

The study progress and findings will be communicated to schools, participants and the public via the trial website, social media and easy to read reports.

## Discussion

This trial is the result of a commissioned call in November 2015 by the National Institute for Health Research, Health Technology Assessment Programme, asking the question: “What is the clinical and cost-effectiveness of a digital behaviour change programme to improve oral health in deprived young people?” The trial was commissioned on the basis of the findings from a study in New Zealand where an increase in self-reported toothbrushing in young adults resulted from the use of a series of motivational SMS [27]. The authors concluded that motivational SMS improved self-reported oral health and further research was needed. They suggested an RCT that included a longer intervention with tailoring of the messages. The protocol for this trial meets the commissioning brief and has used evidence and theory to design a deliverable trial evaluating a complex intervention (Additional file 1).

This trial is the first school-based RCT of an mHealth intervention targeting oral health behaviour in adolescents. A number of interventions have been influential in reducing caries in younger children but adolescents are an often neglected group who also experience the impact of dental caries on their daily lives and into adulthood [44]. There has been an increased interest in the use of digital technology for health improvement with adolescents given the potential to deliver large-scale health behaviour change interventions and the ubiquity of mobile phone use [45]. The results of this study will help to inform evidence-based practice for mHealth interventions aimed at improving oral health. The study will also address limitations of existing studies in that it will have sufficient power to detect change, includes a 3-year follow-up period and dental assessors will be blinded [23–25].

The variety of comprehensive and validated clinical and child-centred measures in this trial is a strength which will help the findings to be compared to, and possibly combined with, other trials investigating interventions to improve children’s oral health. We are looking at the veracity of the self-reported measure of toothbrushing by using two proxy clinical objective indicators (clinically assessed plaque and gingivitis measures using validated tools). Additionally, HRQoL and OHRQoL will be assessed using self-reported data from young people using validated child-centred questionnaires.

Other strengths of the trial include its being conducted across three of the four nations (England, Scotland and Wales), which increases the generalisability of the findings within the UK education systems. We have worked with secondary schools to ensure the intervention fits within the curricula around health and well-being. The SMS message delivery system used can be automated should future roll-out be indicated.

While a further strength of the study is the focus of the recruitment of schools with above the national average percentage of pupils eligible for FSM, this does create challenges. These schools may have limited capacity to cope with the demands of involvement in a RCT while also demonstrating educational improvement of their pupils. For this reason schools judged as having serious weaknesses were excluded.

One of the limitations of this trial is the risk of contamination within schools between one participating year group and the other. We investigated this within the internal pilot trial and found that 27% of the control group pupils had potentially received some of the intervention messages. However, it is highly unlikely that all 27% received the full intervention effect (i.e. received the classroom based session and be receiving bi-daily SMS toothbrushing reminders). The sample size calculation accounts for the possibility of partial contamination effects (i.e. those contaminated gain half the treatment benefits) for 27% of the control group.

If the findings of the trial show that the intervention is effective, this will inform policy to encourage embedding of the intervention into school curricula and the adoption into guidance produced by departments of education and public health and dental organisations.

The BRIGHT trial is currently recruiting schools and young people participants. Recruitment began in October 2017 and will be completed in July 2019.

## Conclusions

- The BRIGHT trial aims to reduce oral health inequalities through use of an mHealth intervention, targeting those young people living in the most deprived areas.
- The findings of the BRIGHT trial have implications for embedding oral health interventions into school curricula guidance produced by national bodies, including departments for education and dental public health and guideline development organisations.

## Additional file

Additional file 1: SPIRIT 2013 checklist: recommended items to address in a clinical trial protocol and related documents. (DOC 121 kb)

## Data Availability

The final anonymised trial data set will be available to all trial team members/investigators if a formal request describing their plans is approved by the trial steering group. To ensure confidentiality, data dispersed to project team members will be blinded of any identifying participant information.

## Abbreviations

BRIGHT: Brushing RemInder 4 Good oral HealTh
CARIES-QC: A measure of child oral health related quality of life
CBS: Classroom-based session
Chilypep: Children and Young People’s Empowerment Project
CHU9D: Child Health Utility-9D –a measure of child health-related quality of life
DMFT: Decayed, missing and filled permanent teeth
FSM: Free school meals
GCSE: General Certificate of Secondary Education qualification taken by secondary school students (equivalent is Scottish Qualifications Authority National 5)
HRQoL: Health-related quality of life
ICC: Intraclass correlation coefficient
ICDAS: International Caries Detection and Assessment System
ICER: Incremental cost-effectiveness ratio
KOB: Keep on Brushing programme—a study of text messaging for unemployed young people in New Zealand
mHealth: Mobile health—describes multimedia technologies that interface with health care delivery and are supported by mobile devices
NICE: National Institute for Clinical Excellence
OHRQoL: Oral health-related quality of life
QALY: Quality adjusted life year
RCT: Randomised controlled trial
SMS: Short messaging service—texting for mobile phones
